# Case Report: Ocular paroxysmal non-epileptic events as the presenting sign of celiac disease in children: a case series

**DOI:** 10.3389/fped.2024.1450563

**Published:** 2024-10-30

**Authors:** Alice Monzani, Giulia Genoni, Amanda Papa, Noemi Paggi, Eleonora Capra, Francesca Brustia, Ivana Rabbone

**Affiliations:** ^1^ Division of Pediatrics, Department of Health Sciences, University of Piemonte Orientale, Novara, Italy; ^2^ Neonatal and Pediatric Intensive Care Unit, Maggiore della Carità University Hospital, Novara, Italy; ^3^ Infantile Neuropsychiatric Unit, Maggiore della Carità University Hospital, Novara, Italy

**Keywords:** celiac disease, children, pediatric, eye movements, neurologic manifestations, pediatric-onset paroxysmal non-epileptic events

## Abstract

**Introduction:**

Neurologic manifestations may be presenting signs of celiac disease (CD). Pediatric-onset paroxysmal non-epileptic events (PNEEs) are not included among them.

**Cases presentation:**

We report the case of two children who presented with ocular PNEEs in association with mild symptoms evocative for CD, who were subsequently diagnosed with CD and experienced regression of PNEEs on a gluten-free diet. Data from 12 patients undergoing neurological evaluation in 2019–2023 for ocular PNEEs were reviewed: 3 (25%) had a subsequent diagnosis of CD.

**Conclusions:**

Ocular PNEEs could be a presenting manifestation of CD. In the diagnostic work-up of PNEEs, screening for CD could be included, both to avoid unnecessary tests and to promptly start a gluten-free diet, which might lead to a favorable clinical response.

## 1 Introduction

Celiac disease (CD) is a systemic, chronic autoimmune disorder triggered by the ingestion of gluten in genetically predisposed individuals. It is characterized by the presence of a variable combination of gluten-dependent clinical manifestations, CD-specific antibodies, HLA-DQ2 or HLA-DQ8 haplotypes, and enteropathy. Its prevalence is increasing and varies from 1% to 2% in the general population (1). Clinical manifestations are extremely variable, from classic gastrointestinal symptoms to a wide range of extra-intestinal manifestations, including various neurological disorders, such as migraine, gluten ataxia, peripheral neuropathy, attention-deficit/hyperactivity disorder, and epilepsy (2, 3). The prevalence of neurological manifestations in children with CD is approximately 6%–10% (4), lower than in adults, and the exact pathogenesis remains uncertain. Up to now, pediatric-onset paroxysmal non-epileptic events (PNEEs) have not been reported among them. PNEEs include a group of disorders manifesting as alterations in motor and/or behavioral activity, beginning abruptly and ending in a short time, which are not determined by neurophysiological dysfunctions, that can mimic and therefore be easily confused with epileptic seizures (5).

Herein, we report the cases of two pediatric patients with ocular PNEEs as the presenting manifestation of CD, and the preliminary results of a small retrospective study of subjects presenting with ocular PNEEs, subsequently diagnosed with CD.

## 2 Cases description

**Figure 1:**
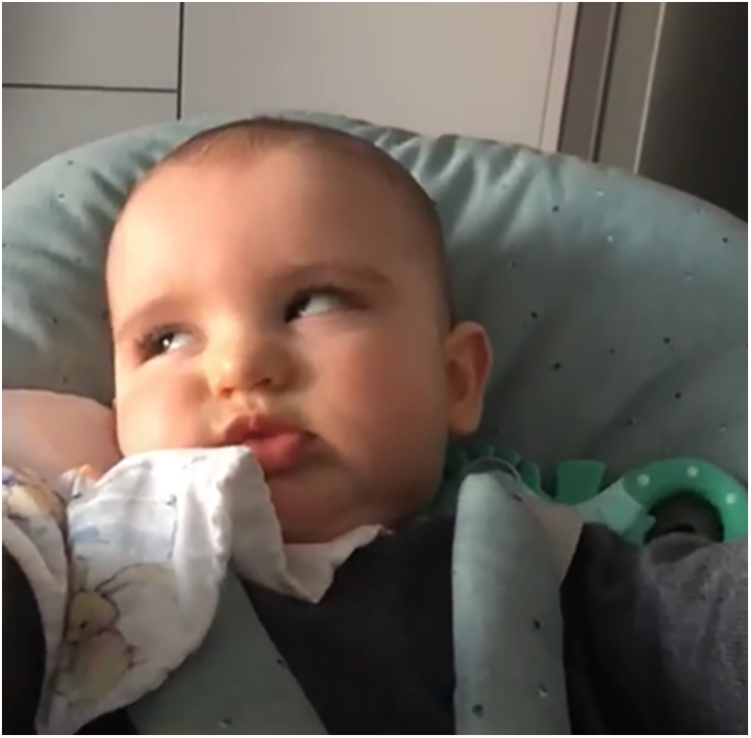
Paroxysmal tonic up-gaze and lateral eye deviation in patient 1.

A male patient aged 2 years and 4 months was referred to our Paediatric Unit due to paroxysmal tonic up-gaze and lateral eye deviation. Such symptoms appeared when he was 7-month old and progressively increased over time (Figure 1). They were preceded by partial eyelid blinking and accompanied by a lip grimace.

The patient had been delivered via elective caesarean section at 38 weeks + 2 days of gestational age due to a transverse presentation, with a birth weight adequate for his gestational age. He was breastfed for the first 12 months, with complementary nutrition introduced at 5 months and gluten at 6 months. During his first year of life, he experienced mild constipation following the introduction of solid foods, which had been managed with Macrogol. His growth in height and weight had remained within normal parameters until the age of 2 when his weight centile had begun to slightly decline.

For paroxysmal eye movements, the patient first underwent neurological and ophthalmological evaluations that were normal. Ictal video-electroencephalography showed no correlates. Routine biochemical tests (liver and kidney function, inflammatory indexes, cytolysis indexes, glucose, and electrolyte levels) were all within the normal range. A blood workup to test a panel of 233 genes associated with neurodevelopmental disorders showed a variant of uncertain significance (c.322G>A, p.Val108Met) in the SLCA1 gene, known to be responsible for GLUT1 deficiency syndrome, whereas no other mutations or variants were detected, in particular for CACNA1A gene, described mutated in paroxysmal tonic upward gaze (6). The SLCA1 analysis was extended to the parents, revealing that the variant was inherited from the healthy father. Therefore, to rule out GLUT1 deficiency syndrome, a lumbar puncture was performed to evaluate the glycemia/glycorrhachia ratio, with negative results. Moreover, a brain magnetic resonance under sedation revealed no abnormalities.

At the age of 2 years and 8 months (25 months after paroxysmal eye movements first appearance), due to a positive family history of CD in a paternal uncle and cousin, anti-transglutaminase antibodies (TG-IgA) were tested, resulting 50× and 20× ULN (upper limit of normal), with positive anti-endomysium IgA, posing a diagnosis of CD without performing biopsies, according to ESPGHAN guidelines. Therefore, a gluten-free diet (GFD) was prescribed. Soon after initiating the GFD, PNEEs frequencies significantly decreased and they almost completely disappeared after 8 months of GFD, when CD serology was repeated, testing negative (TGA-A 0.5 × ULN and EMA IgA negative).

This evolution suggests that PNEEs may have been a manifestation of CD.

**Figure 2:**
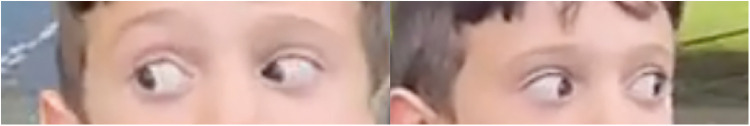
Paroxysmal lateral eye deviation in patient 2.

A 7 year-old boy presented with paroxysmal lateral eye deviation since he was 6 (Figure 2). He was born by a vaginal eutocic delivery at 36 weeks + 6 days of gestational age, with a birth weight adequate for his gestational age. He had been breastfed for the first 24 months, with complementary nutrition (and gluten) introduced at 6 months of age. His growth in height and weight was normal and his past medical history was uneventful. For the paroxysmal eye movements, he underwent neurological evaluation, ictal video-electroencephalography, and brain magnetic resonance, all tested normal, posing a diagnosis of ocular PNEEs. Six months later, for recurrent aphthous stomatitis and loss of appetite, he performed blood tests including TG-IgA, which were increased 8× ULN, with positive anti-endomysium IgA antibodies. He underwent esophagogastroduodenoscopy, diagnostic for CD (Marsh IIIb). After the starting of a GFD, in 15 months no further eye movements were noticed. CD serology, repeated 7 months after the diagnosis, tested negative (TGA-A 0.8 × ULN and EMA IgA negative).

Prompted by such acquisition, we retrospectively reviewed data from 12 patients undergoing neurological evaluation in 2019–2023 in our Infantile Neuropsychiatric Unit for ocular PNEEs. All of them had normal neurophysiology and neuroimaging tests at the time of evaluation. Through a phone interview, we collected that 3 of them (25%) turned out to have a subsequent diagnosis of CD (included the two patients described above), 3 (25%) tested negative for CD serology, and 6 (50%) had never been screened for CD.

## 3 Discussion and conclusions

We reported the cases of two children who presented with ocular PNEEs in association with mild CD-evocative symptoms (constipation and mild failure to thrive in one case, and recurrent aphthous stomatitis and loss of appetite in the other one), subsequently diagnosed with CD. In the younger one, ocular PNEEs appeared soon after gluten introduction and promptly decreased after a GFD initiation; in the older one, PNEEs appeared at the age of 6 years. To our knowledge, it is the first time that ocular PNEEs are described as a possible presenting manifestation of CD.

Hence, abnormal ocular motility in association with CD was previously reported as Gaze-evoked nystagmus and other ocular signs of cerebellar dysfunction in up to 80% of patients with gluten ataxia (7) and as an isolated case report of a 2-year-old boy who presented with CD and opsoclonus-myoclonus syndrome, including action myoclonus, palpebral flutter, opsoclonus, and ataxia (8).

Regarding general neurological manifestations, in adults data suggests that neurologic or psychiatric dysfunction may be the presenting sign of CD in up to 36% of patients (9) and may develop in up to 22% of subjects with diagnosed CD (10), whereas 57% of people with neurological dysfunction of unknown origin tested positive for anti-gliadin antibodies in 1998, before the clinical introduction of antibodies against tissue transglutaminase-2 (11).

In childhood, neurological manifestations have been reported in approximately 6%–10% of patients with CD in a systematic review and meta-analysis (4). According to a more recent community-based cross-sectional study in the UK, up to 28% of children with CD had neurological problems (12), and as much as 51.4% of neurological disorders were described in patients with CD as assessed with a questionnaire (13).

Neurological manifestations described in association with CD include ataxia and cerebellar degeneration, neuropathy, epilepsy, encephalopathy, schizophrenia, depression, migraine, anxiety disorders, attention deficit and hyperactivity disorder, autism, multiple sclerosis, myasthenia gravis, myopathy, and white matter lesions (14).

With regard to the pathogenesis of such manifestations, nutritional deficits, gluten toxicity, and gluten related autoantibodies have been proposed as possible causes. Nutritional deficiencies secondary to malabsorption, have been reported, namely, vitamin B12 deficiency in association with myelopathy and neuropathy, vitamin D deficiency with myopathy, and vitamin E deficiency with ataxia and myopathy (15). Selective neuronal degeneration has also been hypothesized, possibly as a result of an immune response targeting neuronal transglutaminase and other neuronal targets, possibly through a molecular mimesis mechanism (16). Moreover, a role could be played by the dysbiosis known to characterize subjects with CD, altering the gut-brain axis through different possible pathways: via a hormonal network by small molecules, such as lipopolysaccharide, vascular endothelial growth factors, and free radicals; via specific miRNAs up- or down-regulation; via the down-regulation of tight junctions-related proteins, involved in preserving the integrity of both the intestinal barrier and the blood–brain barrier (17).

With reference to the latency time between the onset of neurological symptoms and CD diagnosis, reports in the literature are controversial, as neurological manifestations may sometimes prompt the investigations leading to the diagnosis of CD or, conversely, neurological symptoms may present after the CD diagnosis. For instance, the risk for epilepsy and headache is reported to be increased both before and after CD diagnosis, suggesting a common underlying etiology or predisposition (18, 19). In our patients, PNEEs appearance preceded CD diagnosis, with a latency of 25 months in the first case and about 18 months in the second one. However, this latency may be due to a delay in screening for CD in the absence of other evocative signs or symptoms; accordingly, the delay in CD diagnosis could be reduced if a screening for CD would be included in the initial assessment of PNEEs.

Adherence to a strict GFD can result in clinical improvement in neurologic signs of CD, but this data is controversial (3). GFD seems to have a beneficial effect on gluten neuropathy, however, its benefit on ataxia is less clear, and the response to treatment with a GFD seems to vary according to ataxia duration. Also the latency time between the starting of GFD and neurological manifestations resolution is not easy to determine and its variability may at least in part depend on the degree of GFD adherence. In the patients we described, PNEEs disappeared very quickly after the starting of GFD, that was strictly followed in both cases, as suggested by their serological trends.

In our small retrospective study, 25% of patients evaluated for ocular PNEEs had a subsequent diagnosis of CD. It is conceivable that the proportion of patients with ocular PNEEs and CD could be even higher since TGA-IgA antibodies, commonly used to screen for CD, could be negative in the presence of neurological manifestations of CD. Indeed, in patients without overt gastrointestinal involvement, serum antibodies to transglutaminase-2 (TG2) can be absent, and antibodies primarily reacting with a different transglutaminase isozyme, namely TG6 in patients with neurological manifestations, can be found (14).

The main limitation of this work is the extreme small sample size. However, our preliminary data might pave the way for a pilot study to screen for CD those patients referring to infantile neuropsychiatric units for PNEEs, possibly including anti-TG6 antibodies assessment. After the initial assessment with neurologic evaluation and video-EEG, CD screening might be considered before other more invasive procedures.

If confirmed in larger samples, this data would suggest to screen children with ocular PNEEs for CD, both to possibly avoid useless tests and to readily start a GFD that might produce a favourable clinical response.

## Data Availability

The data analyzed in this study is subject to the following licenses/restrictions: the raw data supporting the conclusions of this article will be made available by the authors, without undue reservation. Requests to access these datasets should be directed to Alice Monzani, alice.monzani@med.uniupo.it.
